# SARS-CoV-2 Vaccine Breakthrough by Omicron and Delta Variants, New York, USA

**DOI:** 10.3201/eid2810.221058

**Published:** 2022-10

**Authors:** Alexander C. Keyel, Alexis Russell, Jonathan Plitnick, Jemma V. Rowlands, Daryl M. Lamson, Eli Rosenberg, Kirsten St. George

**Affiliations:** New York State Department of Health, Albany, New York, USA (A.C. Keyel, A. Russell, J. Plitnick, J.V. Rowlands, D.M. Lamson, E. Rosenberg, K. St. George);; State University of New York, Albany (E. Rosenberg, K. St. George)

**Keywords:** COVID-19, 2019 novel coronavirus disease, coronavirus disease, severe acute respiratory syndrome coronavirus 2, SARS-CoV-2, viruses, respiratory infections, zoonoses, variants, Omicron, Delta, conditional logistic regression, vaccine breakthrough, New York, United States

## Abstract

Recently emerged SARS-CoV-2 variants have greater potential than earlier variants to cause vaccine breakthrough infections. During emergence of the Delta and Omicron variants, a matched case–control analysis used a viral genomic sequence dataset linked with demographic and vaccination information from New York, USA, to examine associations between virus lineage and patient vaccination status, patient age, vaccine type, and time since vaccination. Case-patients were persons infected with the emerging virus lineage, and controls were persons infected with any other virus lineage. Infections in fully vaccinated and boosted persons were significantly associated with the Omicron lineage. Odds of infection with Omicron relative to Delta generally decreased with increasing patient age. A similar pattern was observed with vaccination status during Delta emergence but was not significant. Vaccines offered less protection against Omicron, thereby increasing the number of potential hosts for emerging variants.

As of August 10, 2022, the SARS-CoV-2 pandemic had claimed >6.4 million human lives globally, >1 million in the United States, and >70,000 in New York state (*1*). Virus evolution and adaptation have been observed in persistently infected immunocompromised persons (*2*) and animal reservoirs (*3*,*4*), leading to the potential for new, highly adapted variants.

Novel variants of SARS-CoV-2 have shown increased rates of transmission and immune evasion (*5*,*6*). In particular, Omicron has evolved a suite of unique mutations, which have greatly increased its infectiousness (*7*), increased its ability to evade current vaccines (*5*,*6*), and decreased the effectiveness of convalescent plasma transfusions and monoclonal antibody treatments (*8*,*9*). To a lesser degree, the Delta variant showed some of these same patterns of increased infectiousness (*10*) and potential for immune evasion compared with earlier strains that preceded Delta (*11*).

Prior literature has also shown differences in vaccine effectiveness for SARS-CoV-2 lineages associated with variation in vaccine type, time since vaccination, and patient age. Before emergence of the Delta and Omicron variants, data showed reduced neutralizing antibody protection for the Janssen vaccine (Johnson & Johnson, https://www.jnj.com) compared with the Pfizer (Pfizer-BioNTech, https://www.pfizer.com) and Moderna (https://www.modernatx.com) vaccines (*12*) and slightly stronger protection for Moderna compared with Pfizer vaccines (*12*). An effect of time since vaccination has been demonstrated for the Delta variant (*11*). Younger persons were found to be more likely to be infected with Omicron (*13*,*14*).

To test the associations between vaccination status, vaccine type, and time since vaccination with lineage identity during the emergence of new variants of SARS-CoV-2, we conducted a matched case–control study. We performed analyses for the emergence of the Omicron and Delta variants in New York, USA. The study was waived by the New York State Department of Health (NYSDOH) Institutional Review Board for Human Subjects Research review.

## Methods

### Data Analysis

#### Omicron Emergence Analysis

**Figure 1:**
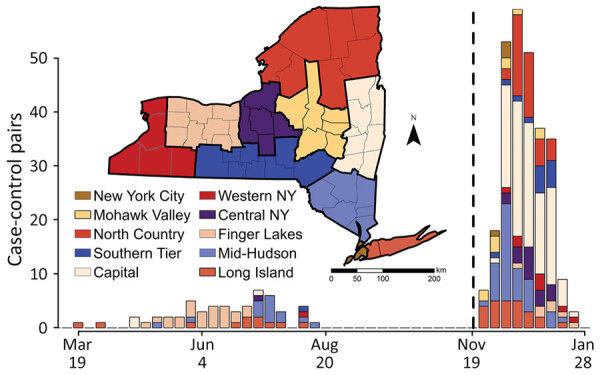
Matched case–control pairs used in the conditional logistic regression by analysis for the SARS-CoV-2 Delta variant (March 19, 2021–August 15, 2021) and the Omicron variant (November 28, 2021–January 24, 2022) emergence periods, by economic region (map), New York, USA. The bars correspond to the order given in the legend; New York City is on top when present and Long Island on bottom when present. The dashed line separates the 2 datasets used in the analyses; the Delta emergence period is on the left and the Omicron emergence period on the right. Map base layer was derived from a combination of 2 public domain layers (US Census data, https://www.census.gov/geo/maps-data/data/tiger-line.html) and Natural Earth Administrative boundaries (https://www.naturalearthdata.com/downloads/50m-cultural-vectors/50m-admin-1-states-provinces).

We analyzed emergence of the SARS-CoV-2 Omicron variant from November 28, 2021, through January 24, 2022 (Figure 1). We matched persons infected with Omicron (case-patients) to persons infected with any other virus lineage (controls). Case-patients (n = 1,439) included infection with B.1.1.529 and all BA sublineages (at the time of the analysis, none were classified as BA.2 through BA.5). Controls (n = 728) were persons infected with all other SARS-CoV-2 lineages circulating during the period of Omicron emergence (all sequenced control samples in the matched dataset were Delta variant, B.1.617.2 or AY sublineages). We defined the start of the Omicron emergence period as the first detection in the genomic surveillance dataset (although Omicron was present in the state before that date). The emergence period ended when the last non-Omicron case was detected in the surveillance dataset. One additional case of infection with Delta was identified >14 days after the last date in the surveillance dataset but was excluded because the sensitivity analysis indicated that it would not substantively change the analysis results. We matched case-patients to controls on the basis of specimen collection date (± 6 days), location (using New York state economic regions [Figure 1]), patient age, and patient sex. We matched age according to age groups: 0–4, 5–11, 12–17, 18–29, 30–49, 50–69, 70–89, and >90 years. If an exact match could not be found, we allowed mismatches for sex. We used 1-to-1 matching, without replacement (i.e., each case-patient was matched to a unique control). We performed matching in 2 stages. In the first stage, we considered all possible matches for each case-patient. To maximize the sample size, we then sorted case-patients such that the case-patients with the fewest possible matches would be matched to controls first. To estimate odds ratios (ORs) and 95% CIs, we performed 3 sets of conditional logistic regressions.

In analysis 1, we included vaccinated and unvaccinated persons. Key variables tested were vaccination status (binary: yes/no), booster status (yes/no), vaccine type (none, Pfizer, Moderna, Janssen), time since last vaccination or booster (3 factor levels: unvaccinated, vaccinated <90 days, vaccinated >90 days). We explored time since completion of initial vaccination and time since booster but found these factors were less predictive and overlapped strongly with the combined time since last vaccination or booster variable and therefore excluded them.

In analysis 2, we examined the association between patient age and virus lineage and therefore removed age as a matching criterion. We performed a conditional logistic regression using age, other main variables for context, and interactions. For this analysis, we did not perform sorting before matching. We examined age in 2 ways: with each age group treated as a factor and with each age group treated as a continuous predictor. Model exploration revealed that a mixture of categorical and continuous predictors best described the underlying data structure (Appendix Table 1).

**Table 1:** Descriptive statistics for matched case-patients and controls for the conditional logistic regression model for study of SARS-CoV-2 vaccine breakthrough during the emergence period of the Omicron variant, New York, USA*

Demographic group	No. (%)							
	Analysis 1, main			Analysis 2, by age			Analysis 3, vaccinated only	
	Controls	Case-patients		Controls	Case-patients		Controls	Case-patients
Age, y								
0–4	4 (1.5)	4 (1.5)		9 (2.9)	4 (1.3)		0	0
5–11	4 (1.5)	4 (1.5)		7 (2.3)	9 (2.9)		0	0
12–17	11 (4)	11 (4.0)		15 (4.9)	16 (5.2)		3 (2.3)	3 (2.3)
18–29	55 (20.2)	55 (20.2)		39 (12.6)	85 (27.5)		23 (17.8)	23 (17.8)
30–49	95 (34.9)	95 (34.9)		85 (27.5)	95 (30.7)		49 (38.0)	49 (38.0)
50–69	71 (26.1)	71 (26.1)		96 (31.1)	69 (22.3)		40 (31.0)	40 (31.0)
70–89	31 (11.4)	31 (11.4)		52 (16.8)	30 (9.7)		14 (10.9)	14 (10.9)
>90	1 (0.4)	1 (0.4)		6 (1.9)	1 (0.3)		0	0
Sex								
M	141 (51.8)	147 (54.0)		155 (50.2)	153 (49.5)		65 (50.4)	77 (59.7)
F	129 (47.4)	123 (45.2)		152 (49.2)	155 (50.2)		63 (48.8)	52 (40.3)
Unknown	2 (0.7)	2 (0.7)		2 (0.6)	1 (0.3)		1 (0.8)	0
Region								
Capital	107 (39.3)	107 (39.3)		121 (39.2)	121 (39.2)		47 (36.4)	47 (36.4)
Central New York	17 (6.2)	17 (6.2)		18 (5.8)	18 (5.8)		10 (7.8)	10 (7.8)
Finger Lakes	7 (2.6)	7 (2.6)		9 (2.9)	9 (2.9)		3 (2.3)	3 (2.3)
Long Island	25 (9.2)	25 (9.2)		27 (8.7)	27 (8.7)		12 (9.3)	12 (9.3)
Mid-Hudson	42 (15.4)	42 (15.4)		47 (15.2)	47 (15.2)		26 (20.2)	26 (20.2)
Mohawk Valley	10 (3.7)	10 (3.7)		18 (5.8)	18 (5.8)		4 (3.1)	4 (3.1)
New York City	4 (1.5)	4 (1.5)		6 (1.9)	6 (1.9)		1 (0.8)	1 (0.8)
North Country	38 (14.0)	38 (14.0)		39 (12.6)	39 (12.6)		20 (15.5)	20 (15.5)
Southern Tier	14 (5.1)	14 (5.1)		16 (5.2)	16 (5.2)		2 (1.6)	2 (1.6)
Western New York	8 (2.9)	8 (2.9)		8 (2.6)	8 (2.6)		4 (3.1)	4 (3.1)
Vaccination status								
Unvaccinated	154 (56.6)	82 (30.1)		175 (56.6)	78 (25.2)		0	0
Vaccinated <90 d	3 (1.1)	4 (1.5)		3 (1.0)	5 (1.6)		2 (1.6)	2 (1.6)
Vaccinated >90 d	115 (42.3)	186 (68.4)		131 (42.4)	226 (73.1)		127 (98.4)	127 (98.4)
Pfizer vaccine	64 (23.5)	113 (41.5)		69 (22.3)	135 (43.7)		64 (49.6)	82 (63.6)
Moderna vaccine	43 (15.8)	66 (24.3)		49 (15.9)	82 (26.5)		48 (37.2)	41 (31.8)
Janssen vaccine	11 (4)	11 (4.0)		16 (5.2)	14 (4.5)		17 (13.2)	6 (4.7)
Unboosted	250 (91.9)	211 (77.6)		281 (90.9)	210 (68.0)		108 (83.7)	88 (68.2)
Boosted <90 d	18 (6.6)	49 (18.0)		25 (8.1)	76 (24.6)		18 (14)	37 (28.7)
Boosted >90 d	2 (0.7)	9 (3.3)		2 (0.6)	20 (6.5)		1 (0.8)	3 (2.3)
Pfizer booster	11 (4.8)	41 (15.4)		13 (4.5)	68 (22.7)		10 (7.8)	33 (25.6)
Moderna booster	9 (3.3)	17 (7)		14 (4.5)	281 (9.4)		9 (7.0)	7 (5.4)

*Presence (case-patient) or absence (control) of Omicron was used as the basis for matching. Janssen vaccine, Janssen/Johnson & Johnson (https://www.jnj.com); Pfizer vaccine/booster, Pfizer-BioNTech (https://www.pfizer.com); Moderna vaccine/booster, Moderna (https://www.modernatx.com).

In analysis 3, we again matched case-patients to controls on the basis of age, but we excluded unvaccinated persons to allow time since last dose (vaccination series or booster) to be treated as continuous variables. Unvaccinated persons could not be included in this analysis because assigning them NA (not applicable) would cause these values to be excluded, and 0 would be an unrealistic value.

We tested leverage by removing each case–control pair sequentially, refitting the model and noting the change in the OR. We selected models by using Akaike information criterion (AIC) scores (*15*,*16*). Models with lower AIC scores have more model support, and models with ΔAIC >2 are generally considered less likely models. Because a more complicated nested model can be within ΔAIC of 2, nested models were required to be within 2 × no. model parameters to be considered tied (*17*). Of note, AIC provides a relative ranking of models but provides no information on the absolute fit of the model. We examined the fit of each model by considering its statistical significance and the OR estimates. When test results were not significant, we examined the magnitude of the OR. More research was deemed necessary if the estimated OR was large enough to be a public health concern but 95% CIs included 1.

We performed all analyses in R 4.1.2 (*18*) by using the package survival for conditional logistic regressions code (https://www.github.com/akeyel/CLR) (*19*,*20*). We created the New York state map in ArcGIS 10.6 (ESRI, https://www.esri.com) by using a 2017 Tiger Shapefile from the US Census Bureau (*21*) and Admin 1 States, provinces 50-m cultural vector shapefile from Natural Earth Data (as of March 18, 2022) (https://www.naturalearthdata.com/downloads/50m-cultural-vectors).

#### Delta Emergence Analysis

We analyzed emergence of the SARS-CoV-2 Delta variant during March 19, 2021–August 15, 2021 (Figure 1). The Delta analyses followed the same methods used for the Omicron analyses but with focal virus lineages (603 case-patients) including B.1.617.2 and all AY sublineages. Nonfocal virus lineages (1,816 controls) were all other lineages circulating during the period of Delta emergence (62% B.1.1.7 and Q.4 Alpha, 20% B.1.526 Iota, 3.5% P.1.X Gamma, 1% B.1.351.X Beta); none of the other non–variant of concern strains (13.7% combined) exceeded 5%. We excluded booster-associated variables because booster doses were not available (Appendix Figure 3). We omitted the vaccinated-only analysis because of low statistical power (n = 12 pairs).

### Power Analysis

Statistical power for conditional logistic regression is nonlinear and depends on estimated probabilities. Although we used multiple conditional logistic regression for the analyses described above, to make the power analyses easier to set up and interpret, we calculated statistical power for univariate logistic regression by using the WebPower package (*22*,*23*) as a simplifying assumption. We examined statistical power to detect an OR of 2 with a sample size of 110 for a range of probability values (0.1–0.9 for the upper probability); we adjusted lower probability to give an OR of 2. We then used the upper probability value with the highest power (0.7) to assess statistical power for ORs of 2, 3, and 4 for sample sizes of 50–350 by increments of 50.

### Data Sources

Respiratory swab specimens that were positive for SARS-CoV-2 by real-time reverse transcription PCR were sent from clinical laboratories across the state for whole-genome sequencing at the NYSDOH Wadsworth Center as part of an enhanced genomic surveillance program. Samples were selected for sequencing on the basis of cycle threshold value and region of patient residence; the goal was full geographic coverage across the state. Sample selection criteria did not change over the course of the study period. We matched samples to demographics in the Communicable Disease Electronic Surveillance System and vaccination records in the New York State Immunization Information System. For persons from whom multiple samples were collected, we included only the earliest collected sample with genome available.

Vaccination status for each person was based on dates of sample collection and administration of vaccines. A person was considered unvaccinated if the sample was collected before any vaccination, vaccinated if the sample was collected >14 days after completion of vaccination (first dose of Janssen, second dose of Pfizer or Moderna vaccine), and boosted if the sample was collected any time after receiving a booster of any vaccine type. We removed from the study persons who were partially vaccinated (sample collected between initial dose and 14 days after vaccination completion, n = 261 [90 with Moderna and 171 with Pfizer vaccine]) and persons who received a greater number of vaccinations than normal. This study does not apply to persons who received a third dose as part of their vaccination series (e.g., potentially immunocompromised persons); these persons were removed from the dataset because of different vaccination history and low sample sizes (58 persons who received a third dose <135 days after their second dose were removed).

### Sequencing Methods

We performed whole-genome amplicon sequencing of SARS-CoV-2 by using a modified version of the Illumina ARTIC protocol (https://artic.network/ncov-2019) with ARTIC V3 primers in the Applied Genomics Technology Core at the Wadsworth Center, as previously described (*24*), and amplified later samples with ARTIC V4 primers. We sequenced samples with particularly low virus titers by using AmpliSeq chemistry on the Ion Torrent S5XL sequencer, as previously described (*25*). 

GISAID (https://www.gisaid.org) accession numbers for sequences are available from https://github.com/akeyel/CLR/blob/main/GISAID_accession_IDs.csv. In that chart, the first column shows the GISAID accession number, and the subsequent columns indicate whether the identification number was used in the respective analyses. Data are coded such that –1 indicates records that were removed before analysis, 0 indicates records that met the basic overall study criteria but were not matched for a particular analysis, and 1 indicates that the record was included in the analysis.

## Results

### Omicron Emergence

**Figure 2:**
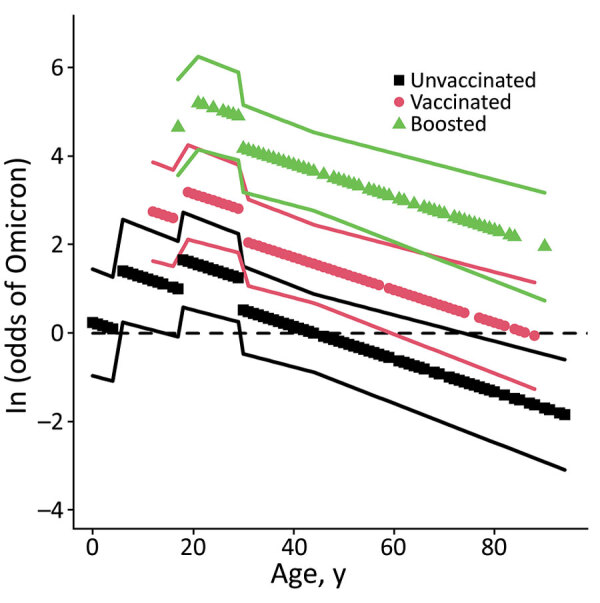
Visualization of the fixed effects from the second Omicron emergence analysis on a log-odds scale (without age matching) in a study of SARS-CoV-2 vaccine breakthrough by Omicron and Delta variants, New York, USA. Odds scale in Appendix. Stratum-specific effects were often strong but were excluded for visual clarity. Increased values indicate an increased probability of infection with Omicron instead of Delta. Lines show ± 1 SE.

In analysis 1, >80% of 272 case-patient/control pairs were 18–69 years of age; most were from the Capital and Mid-Hudson regions (Table 1; Figure 1). Among controls, 8% had received a booster, and among case-patients, 22% had received a booster. Among controls, 56.6% were unvaccinated; among case-patients, 30% were unvaccinated (Table 1). Sample sizes were 177 for Pfizer, 109 for Moderna, and 22 for Janssen vaccine recipients. The variables most associated with an Omicron lineage identity were vaccination (OR 3.1, 95% CI 2.0–4.9; p<0.001) and booster status (OR 6.7, 95% CI 3.4–13.0; p <0.001) (Table 2; Figure 2).

**Table 2:** Variables most associated with an Omicron variant in 3 analyses of SARS-CoV-2 vaccine breakthrough during the emergence period of the Omicron variant, New York, USA*

Model	ΔAIC	Odds ratio (95% CI)		
		Parameter 1	Parameter 2	Parameter 3
Main analysis				
Vaccination status	0	Vaccinated: 3.1 (2.0–4.9)†	Vaccinated + boosted: 6.7 (3.4–13.0)†	
Vaccine type	3.33	Pfizer	Moderna	Janssen
Without booster		3.3 (1.9–5.6)†	3.6 (2.0–6.7)†	2.0 (0.8–5.1)
With booster		10.4 (4.3–25.2)†	3.8 (1.5–9.3)‡	
Age analysis				
Vaccination status + age + age groups	0.00	Vaccinated: 4.8 (2.8–8.1)†	Vaccinated + boosted: 38.5 (15.9–93.2)†	Age, linear: 0.964 (0.950–0.978);† age 0–4 y: 0.250 (0.059–1.051); age 18–29 y: 2.0 (1.1–3.7)§
Vaccination status + age	7.41	Vaccinated: 5.0 (3.0–8.3)†	Vaccinated + boosted: 34.1 (14.6–79.5)†	Age: 0.962 (0.950–0.974)†
Vaccination-only analysis				
Janssen + days after dose	0.00	Janssen, relative to mRNA vaccine: 0.388 (0.149–1.009)		Days after last dose, booster or primary series: 0.996 (0.993–0.999)‡
Vaccine type + days after dose	1.18	Moderna, relative to Pfizer: 0.776 (0.448–1.344)	Janssen, relative to Pfizer: 0.351 (0.132–0.935)§	Days after last dose, booster or primary series:0.996 (0.993–0.999)‡

*Only 1 boosted person had received an initial dose of Janssen vaccine, so for statistical reasons, this person was pooled with the unboosted Janssen recipients. When fit separately, the odds ratio for unboosted Janssen recipients was 1.9 (0.8–4.9) with a parameter p value of 0.20, and the parameter estimate for the single boosted Janssen individual was unreliable. Janssen vaccine, Janssen/Johnson & Johnson (https://www.jnj.com); Pfizer vaccine, Pfizer-BioNTech (https://www.pfizer.com); Moderna vaccine, Moderna (https://www.modernatx.com). ΔAIC, change in Akaike information criterion. †p<0.001. ‡p<0.01. §p<0.05.

In analysis 2 (309 pairs), when patient age was removed as a matching criterion, younger age was also predictive of an Omicron infection; log-odds of infection with Omicron generally decreased as age increased (OR 0.962, 95% CI 0.950–0.974) (Table 2). Significant patterns beyond this log-linear age effect were found for persons in 2 age groups. log-odds of infection with Omicron were lower for persons 0–4 years of age than predicted by a log-linear age effect alone (Figure 2) and higher for persons 18–29 years of age than predicted by a log-linear age term alone; risk was highest for those 18–29 years of age (Figure 2). OR estimates for vaccination status (OR 4.8, 95% CI 2.8–8.1) and booster status (OR 38.5, 95% CI 15.9–93.2) were higher than in the analysis that used age as a matching criterion (Table 2).

In analysis 3 (vaccinated-only persons, 129 pairs), the probability of infection with Omicron decreased with an increased number of days after the last vaccine dose (OR 0.996, 95% CI 0.993–0.999) (Table 2). Vaccine type was also included in the top statistical models (Appendix Table 1) and the trend toward reduced odds of Omicron infection after vaccination with the Janssen vaccine was borderline significant (OR 0.351, 95% CI 0.132–0.935, relative to Pfizer vaccine; OR 0.388, 95% CI 0.149–1.009, relative to any mRNA vaccine) (Table 2).

### Delta Emergence

In analysis 1 (55 pairs), 75% were 18–69 years of age; 89% of case-patients/controls were from the Finger Lakes, Long Island, and the Mid-Hudson regions (Table 3). A total of 74.5% of controls and 61.8% of case-patients were unvaccinated (Table 3). Vaccine type, time from last vaccination, and an interaction of the 2 were not significantly associated with an increased likelihood of infection with Delta than any other virus lineage in the fully matched conditional logistic regression (Table 4). Vaccination status was the top model (OR 2.4, 95% CI 0.8–6.8; p = 0.08). Vaccine type had no significant effect (p = 0.12), but estimated ORs were 2.9 (95% CI 0.9–8.9) for Pfizer, 0.38 (95% CI 0.04–4.2) for Moderna, and 2.0 (95% CI 0.17–23.6) for Janssen.

**Table 3:** Descriptive statistics for matched case-patients and controls for the conditional logistic regression model for study of SARS-CoV-2 vaccine breakthrough during the emergence period of the Delta variant, New York, USA*

Demographic group	No. (%)				
	Analysis 1, main			Analysis 2, age	
	Controls	Case-patients		Controls	Case-patients
Age, y					
0–4	0	0		3 (4.5)	0
5–11	3 (5.5)	3 (5.5)		5 (7.6)	0
12–17	5 (9.1)	5 (9.1)		3 (4.5)	4 (6.1)
18–29	11 (20)	11 (20)		12 (18.2)	10 (15.2)
30–49	26 (47.3)	26 (47.3)		23 (34.8)	30 (45.5)
50–69	6 (10.9)	6 (10.9)		14 (21.2)	17 (25.8)
70–89	4 (7.3)	4 (7.3)		6 (9.1)	5 (7.6)
>90	0	0		0	0
Sex					
M	26 (47.3)	25 (45.5)		28 (42.4)	30 (45.5)
F	29 (52.7)	29 (52.7)		38 (57.6)	35 (53)
Unknown	0	1 (1.8)		0	1 (1.5)
Region					
Capital Region	3 (5.5)	3 (5.5)		4 (6.1)	4 (6.1)
Central New York	1 (1.8)	1 (1.8)		1 (1.5)	1 (1.5)
Finger Lakes	24 (43.6)	24 (43.6)		27 (40.9)	27 (40.9)
Long Island	11 (20)	11 (20)		15 (22.7)	15 (22.7)
Mid-Hudson	14 (25.5)	14 (25.5)		16 (24.2)	16 (24.2)
New York City	0	0		1 (1.5)	1 (1.5)
North Country	0	0		0	0
Southern Tier	1 (1.8)	1 (1.8)		1 (1.5)	1 (1.5)
Western New York	1 (1.8)	1 (1.8)		1 (1.5)	1 (1.5)
Vaccination status					
Unvaccinated	41 (74.5)	34 (61.8)		48 (72.7)	35 (53)
Vaccinated <90 d	7 (12.7)	9 (16.4)		9 (13.6)	9 (13.6)
Vaccinated >90 d	7 (12.7)	12 (21.8)		9 (13.6)	22 (33.3)
Pfizer vaccine	10 (18.2)	18 (32.7)		11 (16.7)	28 (42.4)
Moderna vaccine	5 (9.1)	2 (3.6)		4 (6.1)	3 (4.5)
Janssen vaccine	1 (1.8)	3 (5.5)		3 (4.5)	1 (1.5)

*Presence (case-patient) or absence (control) of Omicron was used as the basis for matching. Janssen vaccine, Janssen/Johnson & Johnson (https://www.jnj.com); Pfizer vaccine, Pfizer-BioNTech (https://www.pfizer.com); Moderna vaccine, Moderna (https://www.modernatx.com).

**Table 4:** Variables most associated with a Delta variant infection in 2 analyses of SARS-CoV-2 vaccine breakthrough during the emergence period of the Delta variant, New York, USA*

Model	ΔAIC	Parameter 1	Parameter 2	Parameter 3
Main analysis				
Vaccination status	0.00	Vaccinated: 2.4 (0.8–6.8)		
Vaccine type	1.05	Pfizer: 2.86 (0.92–8.94)	Moderna: 0.38 (0.04–4.20)	Janssen: 1.97 (0.17–23.57)
Age analysis				
Vaccine type	0.02	Pfizer: 7.3 (2.0–26.7)†	Moderna: 2.0 (0.25–17.1)	Janssen: 0.46 (0.04–4.76)

*None of the main analysis models were statistically significant because all 95% CIs for odds ratio estimates overlapped 1. Janssen vaccine, Janssen/Johnson & Johnson (https://www.jnj.com); Pfizer vaccine, Pfizer-BioNTech (https://www.pfizer.com); Moderna vaccine, Moderna (https://www.modernatx.com). ΔAIC, change in Akaike information criterion. †p<0.01.

The power analysis showed that a sample size of 110 (55 pairs) would have a 15%–45% chance of obtaining a significant result for an OR of 2 under the simulated probability distributions. A sample size of >255 would be needed to have >80% power for an OR of 2. A sample size of 110 could have <78% power to detect an OR of 3 and 93% power to detect an OR of 4. A sample size of 24 could detect an OR of 22 with 80% power but would only have 36% power to detect an OR of 4.

When case-patients and controls were no longer matched on the basis of age (66 pairs), vaccine type was the top model (Appendix Table 2), suggesting that odds of being infected with Delta rather than any other virus lineage increased by a factor of 7.3 (2.0–26.7) for those receiving the Pfizer vaccine relative to unvaccinated persons. Effects for Moderna (2.0, 95% CI 0.25–17.1) and Janssen (0.46, 95% CI 0.04–4.76) vaccines were substantial but not individually significant.

## Discussion

Our exploration of vaccine breakthrough, vaccination status, and time since vaccination in this matched case–control study adds to the body of evidence supporting immune escape of SARS-CoV-2. Some results may seem counterintuitive because of the study design. For example, although a booster increases protection against infection with Omicron compared with absence of a booster (*13*,*26*), history of a booster was associated with Omicron (the emergent strain) and not Delta (the established strain) infection. This finding is consistent with evidence that suggests that having a booster is less effective for preventing infection with Omicron than with Delta (*6*,*13*). Similarly, vaccine effectiveness has been shown to wane with time (*11*); therefore, we hypothesized that increased time after vaccination would decrease the odds of being infected with the emergent strain.

Our analysis of New York state genomic surveillance data yielded results that are consistent with previous research showing an increased probability of breakthrough for Omicron compared with other variants for both vaccinated and boosted persons (*6*,*8*). In a similar study in Connecticut, USA, comparing odds of infection with Omicron versus Delta (*6*), an OR of ≈2 (95% CI 1.5–3.7 or 1.5–2.2, depending on time after vaccination) was found for vaccinated persons and ≈3 (95% CI 1.8–4.9) for boosted persons. These estimates are lower than the estimates from our study of 3.1 (95% CI 2.0–4.9) for vaccinated persons and 6.7 (95% CI 3.4–13.0) for unvaccinated persons, but the 95% CIs overlap between the 2 studies. A strong pattern of the emergent strain shows increased ability for vaccine breakthrough compared with other strains circulating at the time. Studies of prior variants of concern have found significant vaccine breakthrough in emergent variants. For example, Kustin et al. found that vaccine breakthrough for Alpha (B.1.1.7) was more likely compared with prior strains (*27*). Similarly, Tartof et al. found evidence for increased rates of vaccine breakthrough by Delta (B.1.617.2), although waning vaccine immunity was also a factor in that study (*11*). In addition, Rosenberg et al. showed increased breakthrough during the Delta emergence period and suggested that this effect was independent of waning immunity (*28*).

When we restricted the analysis to vaccinated persons only, time after vaccination was a statistically significant factor; probability of Omicron infection decreased with increased time after vaccination. The time-after-vaccination variable combined persons who had recently received a booster with those who had recently completed their primary series. Adding a variable to indicate booster status did not improve the model fit (Appendix Table 1). Of note, most persons in this study were >3 months past completion of their initial vaccination series. Boosters were more recent, and therefore vaccination status and booster status probably encoded much of the same information as a time-after-last-dose variable. No time-after-vaccination effect was detected if the data were coarsely divided into persons who had and had not received boosters, suggesting that more examination of this variable may be necessary. This variable was not found among the top models in the Delta emergence analysis.

Younger persons were more likely to be infected with Omicron than with Delta during the Omicron emergence period, although the data in this study cannot be used to distinguish a physiological basis from a behavioral basis for these age effects. Kahn et al. found Delta and Omicron infection be equally distributed by age among unvaccinated persons but to shift strongly toward younger persons among vaccinated persons (*14*); however, Accorsi et al. found elevated rates of Omicron infection among vaccinated and unvaccinated persons (*13*). It is possible that the age group effects are the result of a greater degree of socialization and other behavioral risk factors among persons 18–29 years of age. In 2020, college campus re-openings were associated with increased transmission of SARS-CoV-2 (*29*). Because Omicron infections can break through vaccinations, college campuses may have increased the likelihood of persons in this age group being infected with SARS-CoV-2 (*30*). The age group effect for preschool children (0–4 years of age) may represent a reduced level of socialization for this group. This effect, although included in the top model identified by the information theoretic approach here, was not statistically significant, so it also may be an artifact of low sample sizes for this age group. Other research has found that vaccines were not equally effective among age groups (V. Dorabawila, unpub. data, https://www.medrxiv.org/content/10.1101/2022.02.25.22271454v1). Vaccine effectiveness in New York was very low for persons 5–11 years of age, who received a lower dose (10 μg) of the Pfizer vaccine than for vaccinated persons >12 years of age who received a 30-μg dose (V. Dorabawila, unpub. data, https://www.medrxiv.org/content/10.1101/2022.02.25.22271454v1). However, the log-linear age effect detected here was not driven by children <12 years of age. When children <12 years of age were removed from the analysis, the estimated OR changed from 0.962 to 0.957 (95% CI 0.944–0.971), suggesting that the magnitude of the effect is greater when young children were removed from the analysis. Larger estimates for vaccination status and booster status were also greater when children <12 years of age were removed from the analysis (vaccination status OR 5.4, 95% CI 3.1–9.7; vaccination plus booster status OR 43.0, 95% CI 17.1–108.5). Vaccination rates and booster rates changed substantially during the study periods as well (Appendix Figures 2, 3), but any resulting biases were probably controlled for by the case–control study design.

Sample sizes were generally too small to detect robust vaccine type effects. The Janssen vaccine showed borderline significantly reduced OR for infection with Omicron relative to the Pfizer vaccine in 1 statistical model (Table 2; Appendix Table 1). This result would be consistent with improved performance against Omicron infection or with worse performance of this vaccine against Delta infection, as has been observed (*28*). Otherwise, OR estimates showed the potential for substantial differences, but overlapping 95% CIs prevent drawing robust conclusions (Table 2; Appendix Table 1).

Statistical power was constrained by the limited emergence periods and the relatively small percentage of viruses from COVID-19 case-patients that were sequenced. For Delta, the emergence period occurred during a time of reduced sequencing, because of low overall incidence during the summer of 2021, when Delta displaced previous strains (Figure 1). For Omicron, a larger sequencing effort was made, but the emergence period was considerably shorter because of the rapid dominance of the Omicron variant (Figure 1). Sample sizes could potentially be increased by expanding the regional scope of the study or incorporating sequencing results from other research laboratories.

We used only 1 matched set for each analysis. However, because case-patients were randomly matched to controls, other matches were possible. This limitation could be overcome by assessing significance with Monte Carlo simulation over the range of possible matches. That said, visual examination of leverage plots based on removing a single pair suggested that the results were generally unlikely to change with the removal of any single data point. The exception is the Delta analysis, in which a change of 1–2 data points would change the overall statistical significance of the results (Appendix Figure 1) without much change in the estimated OR.

In conclusion, this analysis of the emergence of the Omicron and Delta variants in New York, USA, based on sequenced virus identity broadly supports the results of prior studies (*5*–*8*). Vaccines offered less protection against Omicron infection, thereby increasing the number of potential hosts for emerging variants.

Appendix Supplemental results for study of SARS-CoV-2 vaccine breakthrough by Omicron and Delta variants, New York, USA.
