# Supplementary material for: SARS-CoV-2 Vaccine Breakthrough by Omicron and Delta Variants, New York, USA
Source: Emerg Infect Dis. 2022 Oct;28(10):1990–8. doi: 10.3201/eid2810.221058 (PMC9514330; doi:10.3201/eid2810.221058)
Supplement: Appendix — Supplemental results for study of SARS-CoV-2 vaccine breakthrough by Omicron and Delta variants, New York, USA. [file 22-1058-Techapp-s1.pdf]

Article DOI: https://doi.org/10.3201/eid2810.221058

SARS-CoV-2 Vaccine Breakthrough by Omicron and Delta Variants, New York, USA

Appendix

Appendix Table 1. AIC results for the Omicron analyses

Model[1]	Delta AIC2	K	p-value
1) Conditional Logistic Regression (CLR)			
Booster Status + Vaccination Status	0.00[2]	2	<0.001***
Time after Dose	2.37	2	<0.001***
Vaccination Status	4.15	1	<0.001***
Vaccine Type	5.46	3	<0.001***
Full Model	6.19	6	<0.001***
Booster Status	25.41	1	<0.001***
2) No-age matching CLR			
Age + Age Group (18-29) + Age GroupBin (0 - 4) + Vaccination Status + Booster Status	0.00[3]	7	<0.001***
Age + Vaccination Status + Booster Status	7.41	3	<0.001***
Full Model	11.73	7	<0.001***
Time after Dose × Age	29.29	5	<0.001***
Vaccine Type × Age	43.35	7	<0.001***
Vaccination Status + Booster Status	57.67	2	<0.001***
Age	128.36	1	<0.001***
3) Vaccinated-only individuals CLR			
Time after Dose + Janssen	0.00[4]	2	<0.001***
Vaccine Type + Time Post Dose	1.18	3	0.002**
Vaccine Status + Janssen	1.52	2	0.002**
Time after Dose	2.19	1	0.001**
Vaccine Status + Vaccine Type	2.30	3	0.003**
Booster Status	2.68	1	0.002**
Full Model	3.06	4	0.004**
Time after Dose + Booster Status	3.51	2	0.004**
Vaccine Type × Time Post Dose	3.74	5	0.005**
Time after Dose × Booster Status	5.51	3	0.012*
Vaccine Type	6.72	2	0.021*
Janssen	6.92	1	0.019*

* p < 0.05, ** p < 0.01, *** p <0.001.

[1] Results are for 3 separate conditional logistic regression analyses (see methods). For all analyses, each case of Omicron was matched to a non-Omicron control. AIC scores SHOULD NOT be compared across different analyses. × indicates an interaction, while + indicates variables in a model without an interaction term. Full models did not include interaction effects. Best models are indicated in italics.

[2] Minimum AIC score: 330.38, log-likelihood: -163.19, N = 272 pairs.

[3] Minimum AIC score: 285.21, log-likelihood: -137.61, N = 309 pairs.

[4] Minimum AIC score: 168.43, log-likelihood: -82.21, N = 129 pairs.

Appendix Table 2. AIC results for the two Delta analyses

Model	Delta AIC	K	p-value
1) Conditional Logistic Regression (CLR)			
Vaccination Status	0.00[3]	1	0.085
Vaccine Type	1.05	3	0.116
Time Post Dose	1.66	2	0.191
Full Model	2.50	5	0.132
Vaccine Type × Time Post Dose	6.16	7	0.267
2) No-age matching CLR			
Vaccine Type × Age[1]	0.00[4]	7	**0.002****
Vaccine Type	0.02	3	**0.002****
Vaccine Type + Age	1.99	4	**0.005****
Vaccination Status	3.91	1	**0.008****
Time after Dose × Age	4.68	5	**0.014***
Full Model[2]	5.32	6	**0.015***
Vaccination Status × Age	5.97	3	**0.029***
Age	9.75	1	0.261

* p < 0.05, ** p < 0.01.
[1] Model did not converge.
[2] Model converged before all parameters evaluated, some individual parameter estimates should not be used for inference.
[3] Minimum AIC score: 75.3, log-likelihood -36.3, N = 55 pairs.
[4] Minimum AIC score: 82.5, log-likelihood -34.2, N = 66 pairs.

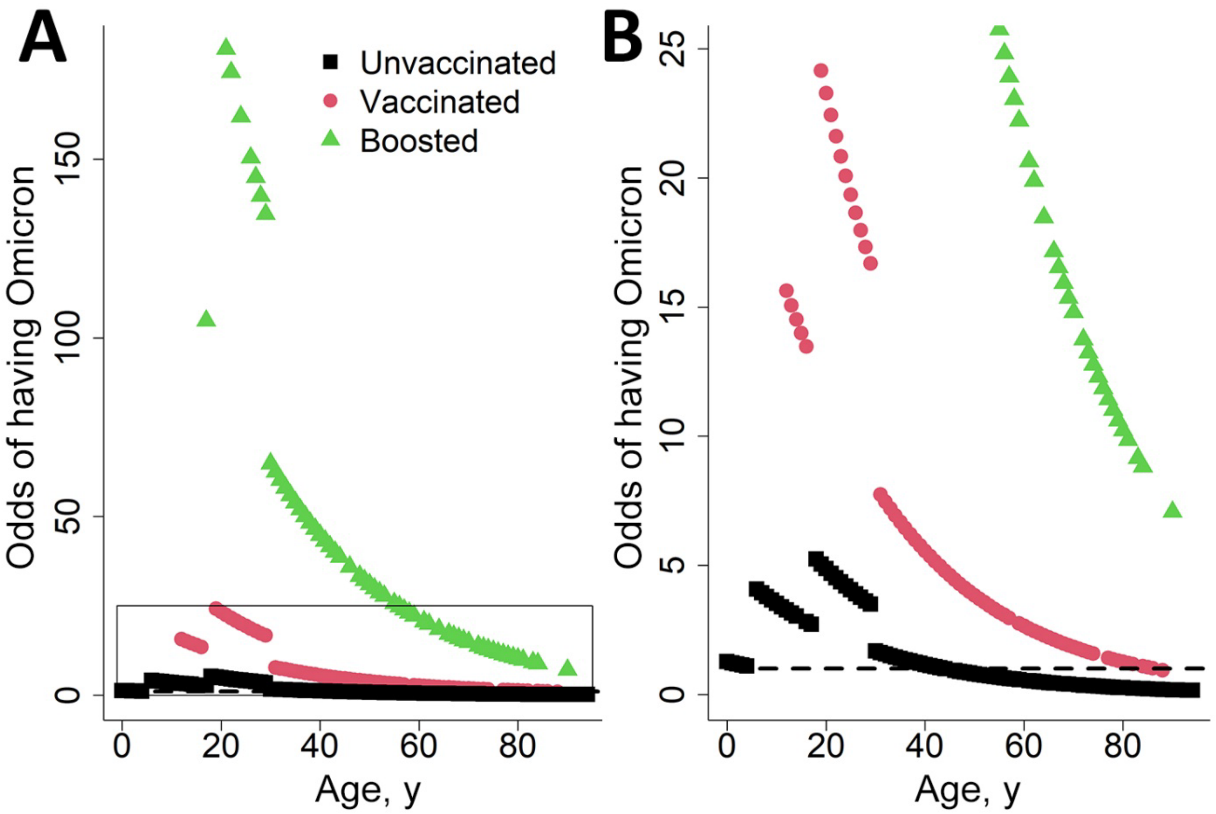

Appendix Figure 1. Visualization of the fixed effects from the second Omicron emergence analysis on an odds scale (without age matching) in a study of SARS-CoV-2 vaccine breakthrough by Omicron and Delta variants, New York, USA. In (A) all data are shown, and (B) shows the data from the box in (A) in greater detail.

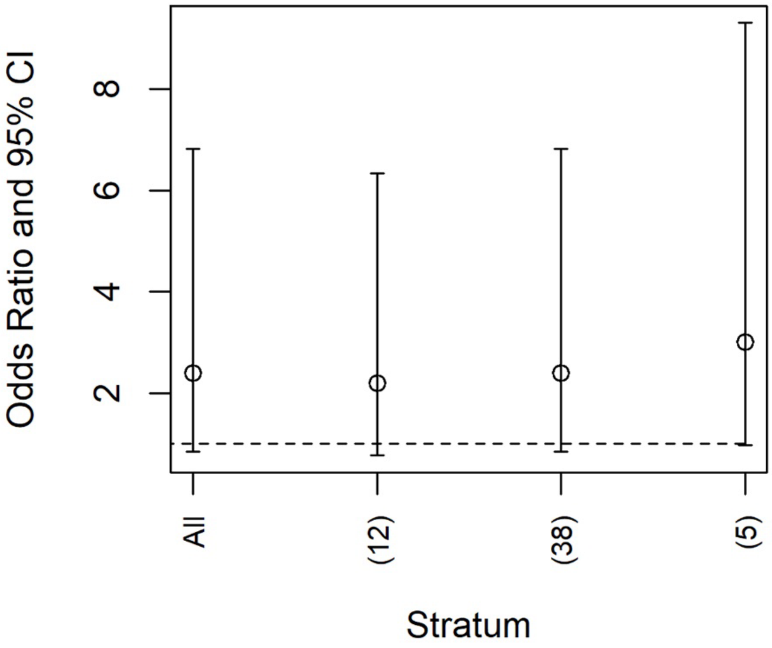

Appendix Figure 2. Primary vaccination rates by age group changed substantially during the Delta Emergence period but increased only slightly during the Omicron Emergence period. The biggest change during the Omicron Emergence period was that the 5 – 11 age group became eligible to become vaccinated. Children under 5 were not eligible to be vaccinated during either emergence period. Vaccination data from the CDC (34)

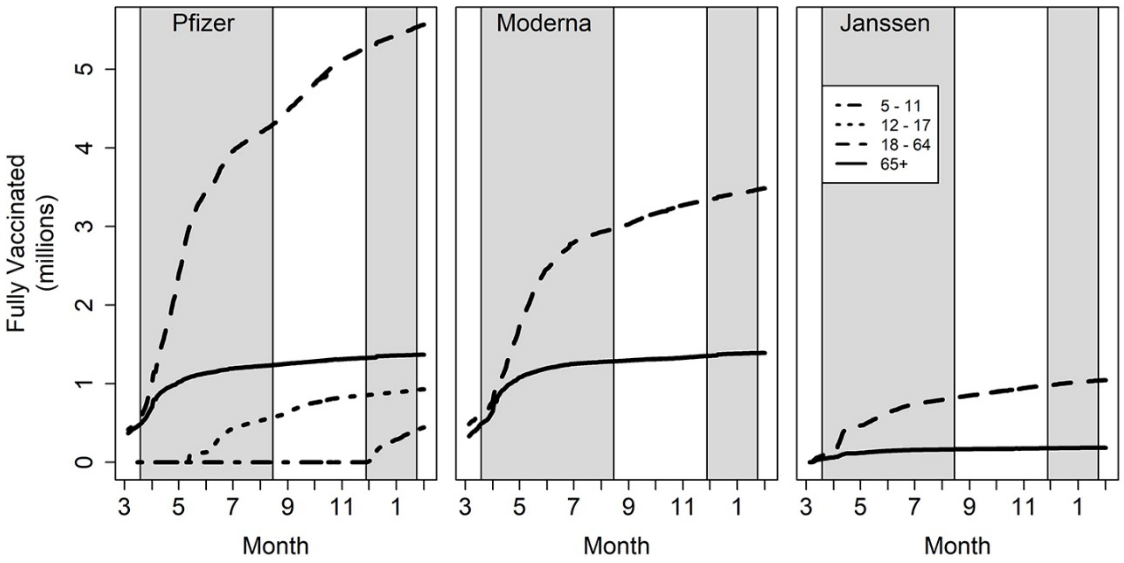

Appendix Figure 3. The number of persons who received a booster greatly increased during the Omicron Emergence period. The general population of 18 – 64-year-olds became eligible to receive boosters during this time period. Boosters were not available during the Delta Emergence period. Vaccination data from the CDC (*1*).

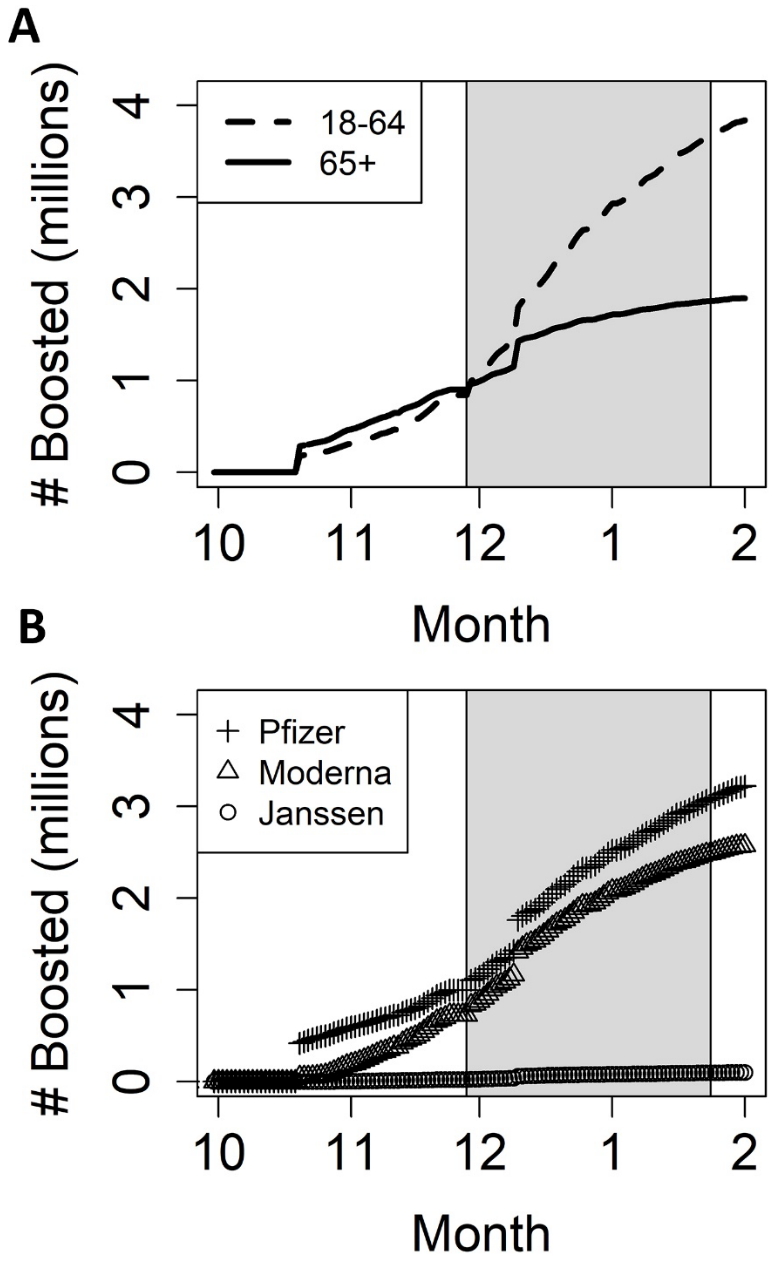

Appendix Figure 4. Leverage plot for the Delta Emergence analysis for the odds ratio for vaccination status. 'All' denotes the model fit including all pairs. The other entries show the model estimated odds ratio and confidence interval if a single pair was removed. If the removal of two pairs had the same estimated effect size, these data points were grouped together, with the number of pairs with similar effects given on the x-axis. For example, the right-most point shows that there were 5 pairs with similar effect sizes. If any one of those pairs were removed, the confidence interval would be approximately at 1. If two of these pairs were removed, the results would likely be statistically significant. That said, there is no scientific basis for removing these pairs from the analysis. The key point is that the statistical significance, but not the median estimate of the odds ratio, was sensitive to the matching process.

Reference

1. Centers for Disease Control and Prevention. COVID-19 vaccinations in the United States, Jurisdiction [cited 2022 Jul 19]. https://data.cdc.gov/Vaccinations/COVID-19-Vaccinations-in-the-United-States-Jurisdi/unsk-b7fc
